# Impact of Anti-Mycotic Drugs on the Osteogenic Response of Bone Marrow Mesenchymal Stem Cells In Vitro

**DOI:** 10.3390/antibiotics13020186

**Published:** 2024-02-15

**Authors:** Tim Niklas Bewersdorf, Jakob Hofmann, Sebastian Findeisen, Christian Schamberger, Thomas Lingner, Ulrike Sommer, Gerhard Schmidmaier, Tobias Grossner

**Affiliations:** 1 Clinic for Trauma and Reconstructive Surgery, Center for Orthopedics, Trauma Surgery and Paraplegiology, University Hospital Heidelberg, 69120 Heidelberg, Germany; timniklas.bewersdorf@med.uni-heidelberg.de (T.N.B.); jakob.hofmann@med.uni-heidelberg.de (J.H.);; 2 Genevention GmbH, Rudolf-Wissell-Str. 28A, 37079 Göttingen, Germany

**Keywords:** fungicide, anti-fungal drug, Voriconazole, liposomal Amphotericin B, Fluconazole, drug-loaded Polymethylmethacrylate cement, osteogenesis, human bone marrow mesenchymal stem cells, in vitro, ^99m^Technetium-Hydroxydiphosphonate labeling

## Abstract

The treatment of fungal bone infections and infected non-unions is a huge challenge in modern trauma and orthopedics, which normally contain the local and systemic administration of anti-fungal drugs. Although frequently used, little is known about the impact of systemic and locally administered fungicides on the osteogenic regenerative capabilities of infected bone tissue, especially upon the osteogenesis of human bone marrow mesenchymal stem cells (BM-hMSCs). This study evaluates the effects of the three most common fungicides for the systemic treatment of bone infections, Voriconazole (VOR), liposomal Amphotericin B (LAMB), and Fluconazole (FLU), as well as the effects of VOR and LAMB-loaded Polymethylmethacrylate (PMMA) cement chips in different concentrations upon the osteogenic response of BM-hMSCs in vitro. Within this study, we compared the ability of BM-hMSC to differentiate into osteoblast-like cells and synthesize hydroxyapatite as assessed by radioactive ^99m^Technetium-Hydroxydiphosphonate (^99m^Tc-HDP) labeling, cell proliferation, and analyses of supernatants upon various osteogenic parameters. Our results revealed that VOR added to the cell culture medium affects the osteogenic potential of BM-hMSC negatively, while there were no detectable effects of LAMB and FLU. Moreover, we showed dose-dependent negative effects of high- and extended-dose fungicide-loaded PMMA cement due to cytotoxicity, with a higher cytotoxic potential of VOR than LAMB, while low-dose fungicide-loaded PMMA had no significant effect on the osteogenic potential of BM-hMSC in vitro.

## 1. Introduction

Bone infections are one of the most serious complications in modern orthopedic and trauma surgery, and they present a huge challenge for orthopedic surgeons [1,2,3,4]. While most infections are caused by bacteria, there are a small number of fungal infections [2,5,6,7]. Infections with fungi are often devastating for the patient and very complicated to treat because of their high virulence, the limited possibilities of being attacked by drugs, their resistance toward these drugs, and their limited clinical treatment experience, which together often leads to a high morbidity [2,5].

In elective total joint arthroplasty, prosthetic joint infections are the major complication after total knee arthroplasty and the third most common long-term complication in total hip arthroplasty [1,7,8,9]. Infections are even the second most frequent reason for a revision within the first 5 years after total hip arthroplasty [1]. While most infections are caused by bacteria, fungal infections are accountable for 1% of all prosthetic joint infections, and they often require multiple surgeries with a temporary removal of the prosthesis to cure the infection, if possible at all [6,7,10]. Nevertheless, re-infections are common, and they lead to even higher mortality rates [6,7]

Fungal infections of fractures are less prevalent than fungal infections in total joint arthroplasty [11]. The most frequent cause of fracture infections is the direct inoculation of open fractures or traumatic injuries contaminated with soil [12,13,14]. Although fungal fracture infections are very rare, the risk of fungal infection rises with the complexity of injuries [11,15]. Invasive fungal infections of severe injuries of the lower extremities were reported with rates as high as 8% in military trauma intensive care units (ICUs), and 3.5% of all combat injuries and often result in deep wound infections or osteomyelitis [13,16]. Patients with invasive fungal infections have higher rates of amputation, invasive revision therapies, and fracture healing times, as well as at least a higher mortality rate [13,14,15].

If a prosthetic joint infection or a fracture-related infection is confirmed, the correct classification and identification of the microorganism are the most important. As a consequence, the acquisition of histopathological and microbiological probes is very important in order to achieve correct anti-microbiological treatment [3]. The most frequently found fungal pathogens of bone and joint infections are Candida species [6,7,10,12]. Besides Candida species, Aspergillus species is the second most common pathogen in fungal prosthetic joint infections and fungal fracture infections [7,12,13].

The successful treatment of fungal prosthetic joints or fracture infections is predominantly similar and combines surgical and pharmacological therapies [11,12,13]. This combined treatment is necessary because fungi like Candida albicans form biofilms [7,12]. Biofilm is one of the most important virulence factors of fungi because it is commonly associated with anti-fungal resistance, or higher minimal inhibitory concentrations caused by less effective penetration of fungicides [6,7,12,17]. High-dose systemic antimycotic drug treatment without surgical intervention would lead to the suppression of clinical symptoms of infection at the expense of potential toxic side effects without the successful treatment of the infection [7]. Surgical treatment of fungal infections normally requires multiple operations [7,10,11,13]. The goals of the first surgery are to eliminate fungus and retrieve multiple probes to determine the correct species [7,10,13]. Elimination of the fungus contains removal of all remaining foreign bodies and implants, the debridement of necrotic, devascularized, and infected tissue, and the lavage and drainage of fluid collections [7,10,13]. If there is a positive result from fungal infections, most patients receive a second surgery with debridement and lavage [6]. Although systemic anti-fungal drug therapy can lead to severe side effects, like nephrotoxicity, hepatotoxicity, or visual disturbance, it is very important to treat the possible remaining fungi after surgical debridement [12,18].

Besides systemic fungicide therapy, local anti-fungal drug therapy has become more common over the past few years [18,19]. Local anti-fungal drug therapy can deliver exceptional high drug concentrations at the infection site without leading to severe systemic side effects [12,18,19,20]. Recent data confirmed the positive effects of the admixture of anti-fungal drugs into Polymethylmethacrylate (PMMA) bone cement to deliver local, exceptional high drug concentrations at the infection site without leading to systemic side effects [12,18,19,20]. Over the past few years, several different fungicides have been tested for local therapy [6,7,12,17,18,19,20,21,22,23,24,25]. While in most studies, PMMA was used to deliver the fungicide to the infection side [6,7,12,17,19,23,24], some studies used, e.g., biodegradable rods [25], calcium sulfate beads [17,24], or hydroxyapatite [17]. Especially anti-fungal drug-loaded PMMA spacers can provide high doses of fungicide on the infection side for several weeks [12,17]. The admixed dosages of antimycotic drugs in PMMA are based on mechanical strength and anti-fungal effects but not on osteogenic responses or toxicity to the surrounding tissue [18,26,27]. Regarding these mechanical and anti-fungal characteristics, the PRO-IMPLANTAT FOUNDATION recommends low-dose antimycotic drug-loaded fixation cement for the fixation of prothesis and compound osteosynthesis and high-dose antimycotic drug-loaded spacer cement for temporary use [21,22]. Although frequently used, little is known about the impact of these high antimycotic drug concentrations on the osteogenic regenerative capabilities of bone tissue, in particular upon the osteogenesis of human bone marrow mesenchymal stem cells (BM-hMSC). In the past, several therapeutic drug monitoring studies have revealed that most negative effects are dose-dependent [28,29,30,31]. For successful fracture healing, s, non-union healing, or stable incorporation of prothesis it is crucial that these high concentrations do not impair the osteogenic potential of osteoblasts and stem cells on the infection side.

This study evaluated the effects of anti-fungal drugs in simulated, therapeutic local and systemic administration on the ability of BM-hMSCs to differentiate into osteoblast-like cells and synthesize hydroxyapatite (Ca_10_(PO_4_)_6_(OH)_2_) [32] in a monolayer cell culture. In our first step (Part I), we compared the three most common systemic anti-fungal drugs, Voriconazole (VOR), liposomal Amphotericin B (LAMB), and Fluconazole (FLU), each in a low-dose and high-dose concentration added to the cell culture media regarding their effects on the osteogenic potential of human bone-marrow-derived mesenchymal stem cells (BM-hMSC) in vitro. Low- and high-dose concentrations were chosen according to measured serum and tissue concentrations in other studies. VOR: low-dose: 2 µg/mL, high-dose: 5 µg/mL [28,29,32,33,34], LAMB: low-dose: 23 µg/mL, high-dose: 43 µg/mL [28,33,34,35,36], FLU: low-dose: 9 µg/mL, high-dose: 140 µg/mL [23,31,33,37]. In our second step (Part II), we investigated the ability of BM-hMSC to differentiate into osteoblast-like cells and synthesize hydroxyapatite in vitro in conjunction with fungicide-loaded PMMA cement chips in different fungicide concentrations, diluting the drug into the cell culture media. We used VOR and LAMB, which are the only fungicides with approval from the U.S. Food and Drug Administration (FDA) and European Medicines Agency (EMA) for use in PMMA cement at different concentrations. According to the guidelines of PRO-IMPLANT FOUNDATION, we used the recommended low-dose fixation (5 mg of fungicide per g PMMA) and high-dose spacer (10 mg of fungicide per g PMMA) concentrations and an additional extended-dose experimental spacer (15 mg of fungicide per g PMMA) concentration of the fungicide. Although PMMA is frequently used in orthopedic and trauma surgeries, the literature about the effects of PMMA on osteogenic potential and cytotoxicity is controversial [38,39,40,41,42,43,44]. In order to provide more results about this topic, another aim of this study was to evaluate whether and how PMMA cement solely affects osteogenic potential and cell count.

There are several markers to assess the osteogenic potential in vitro. For a precise evaluation of the osteogenic potential, we analyzed supernatants of the cells for concentrations of the osteogenic markers calcium, phosphate, and alkaline phosphatase (ALP). Besides these indirect methods, we performed the novel, direct, non-destructive ^99m^Technetium-Hydroxydiphosphonate (^99m^Tc-HDP) labeling to assess the osteogenic potential, which is defined by the amount of hydroxyapatite produced [45,46]. The radioactive tracer ^99m^Technetium (^99m^Tc) bound to Hydroxydiphosphonate (HDP) directly binds to newly formed hydroxyapatite and can be quantified by an activimeter [46]. This method is a non-destructive way to measure the osteogenic potential of cells, and consequently, cell cultures remain intact for further assessment [47]. To evaluate the effects of anti-mycotic drugs on cell amounts, we performed DAPI staining and automated digital cell counts.

## 2. Results

As mentioned in the introduction, this study was performed in two parts. Every result was normed for 10,000 cells in order to evaluate the osteogenic potential of a normed number of cells.

### 2.1. Part I

#### 2.1.1. DAPI Cell Count

**Figure 1:**
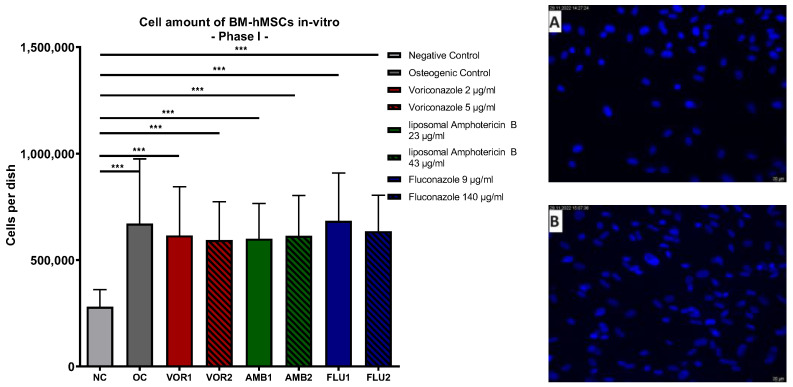
Cells per dish: Mean cell amount per group of BM-hMSCs in vitro in Part I assessed by DAPI staining. Fungicides were added to cell culture medium in the concentration displayed on the right side. The error bars show the error of the mean. Significances are indicated by stars; bars under the stars indicate the compared group for each significance. n ≥ 5. ***: *p* < 0.001. Picture (**A**) displays an example picture of a DAPI microscopy of the negative control and picture (**B**) displays the osteogenic control.

Our analysis of the DAPI cell counts of the samples revealed significantly higher cell counts in all osteogenic groups in comparison to the negative control (*p* < 0.001), without any significant difference between all of the osteogenic group. The negative control (NC) showed the lowest total cell count per dish, while the highest average cell amount was detected in Fluconazole 9 µg/mL (FLU1). The results are displayed in Figure 1.

#### 2.1.2. ^99m^Tc-HDP Labeling

**Figure 2:**
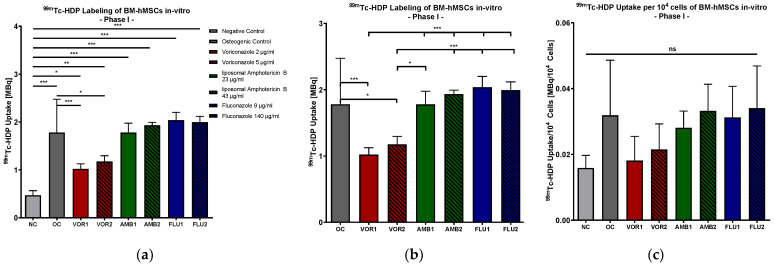
(**a**) ^99m^Tc-HDP uptake in MBq: mean ^99m^Tc-HDP uptake per group of BM-hMSCs in vitro in Part I. (**b**) ^99m^Tc-HDP uptake in MBq: mean ^99m^Tc-HDP uptake per osteogenic differentiation group of BM-hMSCs in vitro in Part I. (**c**) ^99m^Tc-HDP uptake in MBq per 10^4^ cells: mean ^99m^Tc-HDP uptake per group of BM-hMSCs in vitro in Part I was normed for 10^4^ cells. Fungicides were added to cell culture medium in the concentration displayed in the legend. The error bars show the error of the mean. Significances are indicated by stars; bars under the stars indicate the compared group for each significance. n ≥ 5. ns: not significant, *: *p* < 0.05, **: *p* < 0.01, ***: *p* < 0.001.

The mean values of the tracer uptake reflected statistically significant osteogenic differentiation in all of the groups except in the NC (0.469 MBq), as displayed in Figure 2a. Significance was between *p* < 0.05 (NC vs. Voriconazole 2 µg/mL (VOR1)) and *p* < 0.001 (NC vs. osteogenic control (OC)/liposomal Amphotericin B 23 µg/mL (AMB1)/liposomal Amphotericin B 43 µg/mL (AMB2)/Fluconazole 9 µg/mL (FLU1)/Fluconazole 140 µg/mL (FLU2)). The lowest uptake of all osteogenic groups was detected in low-dose VOR (VOR1) (1.022 MBq) and high-dose VOR (5 µg/mL, VOR2) (1.175 MBq), while the highest technetium uptake was registered in low-dose FLU (FLU1) (2.038 MBq) and high-dose FLU (FLU2) (1.995 MBq). ^99m^The Tc-HDP uptake in LAMB groups was 1.780 MBq in the low-dose group (AMB1) and 1.934 MBq in the high-dose group (AMB2).

Comparing the osteogenic differentiation groups with each other, ^99m^Tc-HDP uptake was significantly lower in both VOR groups than in OC (OC vs. VOR1: *p* < 0.001; OC vs. VOR2: *p* < 0.05). In contrast, there was no significant difference between all of the other antimycotic drug groups and OC. Figure 2b.

#### 2.1.3. ^99m^Tc-HDP Labeling Normed for 10,000 Cells

Subsequently, the ^99m^Tc-HDP uptake results of the groups were normed to 10^4^ cells in each dish to evaluate the osteogenic potential of a normed number of cells. Normation was performed to distinguish whether higher ^99m^Tc-HDP uptake results were due to higher cell amounts or higher osteogenic potential per cell. Low-dose and high-dose VOR showed a slightly but not significantly lower ^99m^Tc-HDP uptake per cell than all of the other osteogenic groups. Moreover, there was no significant difference between every group detected, with a slightly higher ^99m^Tc-HDP uptake per cell in the osteogenic groups in comparison to NC. The results are displayed in Figure 2c.

### 2.2. Part II

#### 2.2.1. DAPI Cell Count

**Figure 3:**
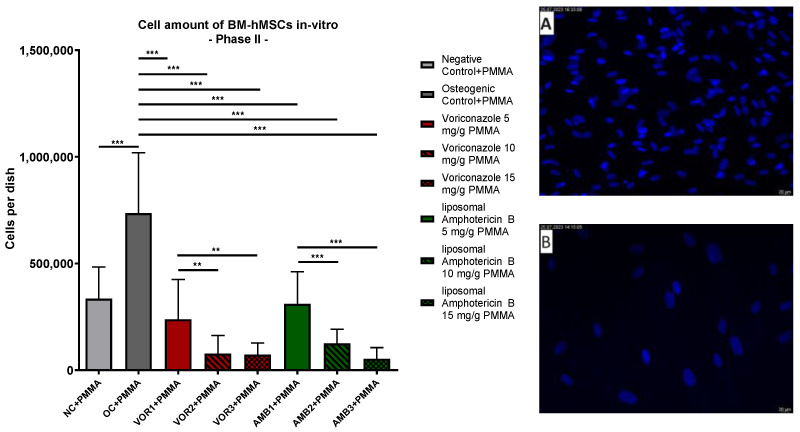
Cells per dish: mean cell amount per group of BM-hMSCs in vitro in Part II assessed by DAPI staining. A 0.5 g PMMA cement chip was placed in the center of every dish. Fungicides were admixed to PMMA in the concentrations displayed on the right side. The error bars show the error of the mean. Significances are indicated by stars; bars under the stars indicate the compared group for each significance. n ≥ 4. **: *p* < 0.01, ***: *p* < 0.001. Picture (**A**) displays an example picture of DAPI microscopy of osteogenic control + PMMA and picture (**B**) displays an example picture of liposomal Amphotericin B 15 mg/g PMMA.

DAPI staining of Part II dishes revealed significantly lower cell counts in every fungicide sample in comparison to the osteogenic control with PMMA chips (OC + PMMA) (*p* < 0.001). The highest average cell count was detected in OC + PMMA (735,881 cells per dish). The average cell amount in OC + PMMA was significantly higher (*p* < 0.001) than the average cell amount in negative control dishes (NC + PMMA) (335,133 cells per dish). With rising VOR concentrations, the average cell amount per dish dropped consistently: 5 mg VOR/g PMMA (VOR1 + PMMA): 238,257; 10 mg VOR/g PMMA (VOR2 + PMMA): 77,718; 15 mg VOR/g PMMA (VOR3 + PMMA): 72,691. These lower cell amounts in VOR2 + PMMA and VOR3 + PMMA were significantly lower compared to VOR1 + PMMA (*p* < 0.01). Similar to these results, the average cell counts per dish dropped significantly with higher LAMB concentrations: 5 mg LAMB/g PMMA (AMB1 + PMMA): 310,774; 10 mg LAMB/g PMMA (AMB2 + PMMA): 124,986; 15 mg LAMB/g PMMA (AMB3 + PMMA): 53,313. Comparing AMB1 + PMMA with AMB2 + PMMA, respectively, AMB3 + PMMA cell counts showed significant differences (*p* < 0.001). No differences between VOR and LAMB in the same concentration were detected. The results are displayed in Figure 3.

#### 2.2.2. ^99m^Tc-HDP Labeling

**Figure 4:**
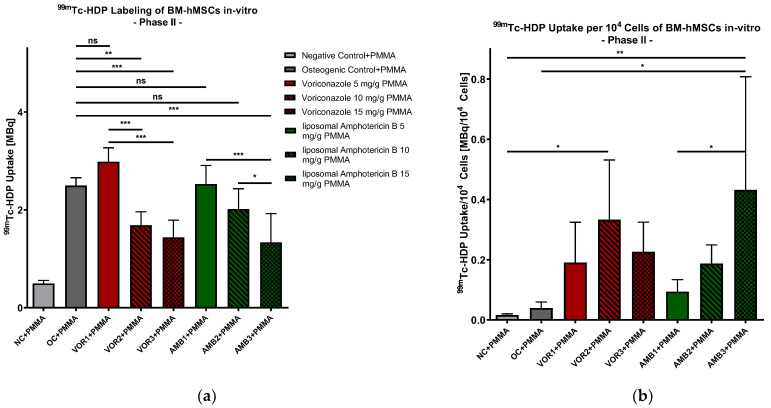
(**a**) ^99m^Tc-HDP uptake in MBq: mean ^99m^Tc-HDP uptake per group of BM-hMSCs in vitro in Part II. n = 6. (**b**) ^99m^Tc-HDP uptake in MBq per 10^4^ cells. Mean ^99m^Tc-HDP uptake per group of BM-hMSCs in vitro in Part II was normed for 10,000 cells. A 0.5 g PMMA cement chip was placed in the center of every dish. n ≥ 4. Fungicides were admixed to PMMA in the concentrations displayed in the middle. The error bars show the error of the mean. Significances are indicated by stars; bars under the stars indicate the compared group for each significance. ns: not significant, *: *p* < 0.05, **: *p* < 0.01, ***: *p* < 0.001.

The mean values of ^99m^Tc-HDP uptake reflected a solid and statistically significant osteogenic differentiation in all of the groups except in NC + PMMA (0.496 MBq). *p*-value varies between *p* < 0.01 (NC + PMMA vs. AMB3 + PMMA) and *p* < 0.001 (NC + PMMA vs. OC + PMMA/VOR1 + PMMA/VOR2 + PMMA/AMB1 + PMMA/AMB2 + PMMA). The highest uptake was measured in low-dose VOR (VOR1) (2.983 MBq), followed by low-dose LAMB (AMB1) (2.527 MBq) and OC (2.496 MBq). With an increasing fungicide concentration, the ^99m^Tc-HDP uptake decreased in all groups. High-dose VOR (VOR2 + PMMA) (1.687 MBq) and extended-dose VOR (VOR3 + PMMA) (1.436 MBq) groups had a significantly lower ^99m^Tc-HDP uptake than VOR1 + PMMA (both *p* < 0.001). Extended-dose LAMB (AMB3 + PMMA) (1.337 MBq) showed a significantly lower ^99m^Tc-HDP uptake compared to low-dose LAMB (AMB1 + PMMA) (*p* < 0.001) and high-dose LAMB (AMB2 + PMMA) (2.017 MBq, *p* < 0.05). Comparing AMB1 + PMMA with AMB2 + PMMA, no significant difference was detected. Our results revealed no significant difference between OC + PMMA and VOR1 + PMMA; AMB1 + PMMA, and AMB2 + PMMA are displayed in Figure 4a.

#### 2.2.3. ^99m^Tc-HDP Labeling Normed for 10,000 Cells

Similar to Part I, the ^99m^Tc-HDP uptake of every group was normed to 10^4^ cells, as displayed in Figure 4b. In comparison to NC + PMMA, there was a significantly higher ^99m^Tc-HDP uptake per cell detected in VOR2 + PMMA (*p* < 0.05) and AMB3 + PMMA (*p* < 0.01). AMB3 + PMMA also showed a significantly higher ^99m^Tc-HDP uptake per 10.000 cells than OC + PMMA (*p* < 0.05) and AMB1 + PMMA (*p* < 0.05). In general, we recognized slightly higher ^99m^Tc-HDP uptake rates per cell with increasing LAMB concentrations. ^99m^The Tc-HDP uptake per cell showed a bell-shaped pattern under increasing VOR concentrations.

#### 2.2.4. Calcium Concentration in Supernatants

**Figure 5:**
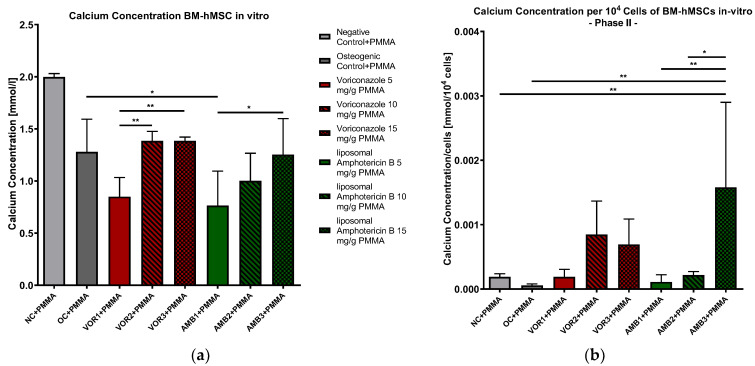
(**a**) Calcium concentration in supernatants of BM-hMSC in vitro. Mean calcium concentration per group of BM-hMSCs in vitro in Part II in mmol/L. n = 6. (**b**) Calcium concentration per 10^4^ cells in supernatants of BM-hMSC in vitro. Mean calcium concentration per group of BM-hMSCs in vitro in Part II normed per 10^4^ cells. n ≥ 4. A 0.5 g PMMA cement chip was placed in the center of every dish. Fungicides were admixed to PMMA in the concentrations displayed in the middle. The error bars show the error of the mean. Significances are indicated by stars; bars under the stars indicate the compared group for each significance. *: *p* < 0.05, **: *p* < 0.01.

The results of the supernatant analyses showed significantly lower calcium concentrations in every osteogenic group compared to NC + PMMA. The *p*-values vary between *p* < 0.05 (NC + PMMA vs. VOR2 + PMMA/VOR3 + PMMA) and *p* < 0.001 (NC + PMMA vs. VOR1 + PMMA/AMB1 + PMMA/AMB2 + PMMA/AMB3 + PMMA). Significant differences in the calcium concentrations in comparison to OC + PMMA were only detected in AMB1 + PMMA (*p* < 0.05). We measured significantly higher calcium concentrations in VOR2 + PMMA and VOR3 + PMMA compared to VOR1 + PMMA (*p* < 0.01) and AMB3 + PMMA compared to AMB1 + PMMA (*p* < 0.05). The calcium concentrations are displayed in Figure 5a.

The results were normed for 10^4^ cells, and a further analysis was performed (Figure 5b). The results clearly showed a significantly higher calcium concentration per 10^4^ cells in AMB3 + PMMA than NC + PMMA (*p* < 0.01), OC + PMMA (*p* < 0.01) and AMB1 + PMMA (*p* < 0.01) and AMB2 + PMMA (*p* < 0.05). Although the calcium concentrations per cell rose with higher LAMB concentrations, further significant differences were not detected.

#### 2.2.5. Phosphate Concentration in Supernatants

**Figure 6:**
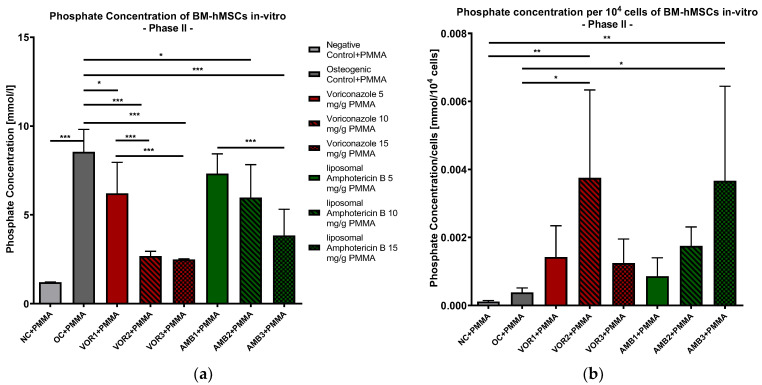
(**a**) Phosphate concentration in supernatants of BM-hMSC in vitro. Mean phosphate concentration per group of BM-hMSCs in vitro in Part II in mmol/L. n = 6. (**b**) Phosphate concentration per 10^4^ cells in supernatants of BM-hMSC in vitro. Mean phosphate concentration per group of BM-hMSCs in vitro in Part II normed per 10^4^ cells. n ≥ 4. A 0.5 g PMMA cement chip was placed in the center of every dish. Fungicides were admixed to PMMA in the concentrations displayed in the middle. The error bars show the error of the mean. Significances are indicated by stars; bars under the stars indicate the compared group for each significance. *: *p* < 0.05, **: *p* < 0.01, ***: *p* < 0.001.

The lowest phosphate concentrations were detected in NC + PMMA with 1.203 mmol/L, while every osteogenic group showed higher phosphate concentrations, with the highest phosphate concentration in OC + PMMA (8550 mmol/L). In general, the phosphate concentration decreased with higher fungicide concentrations in both the VOR and LAMB groups. The phosphate concentration was significantly lower in every VOR group compared to OC + PMMA (VOR1 + PMMA: *p* < 0.05, VOR2 + PMMA and VOR3 + PMMA: *p* < 0.001). Comparing LAMB groups with OC + PMMA revealed significantly lower phosphate concentrations in AMB2 + PMMA (*p* < 0.05) and AMB3 + PMMA (*p* < 0.001) but not in AMB1 + PMMA. Comparing the VOR and LAMB groups with each other, the LAMB groups always showed higher concentrations than the corresponding VOR group. The results are displayed in Figure 6a.

After calculating the quotients of phosphate concentrations per 10^4^ cells, we detected the highest quotients in VOR2 + PMMA (0.00375 mmol/10^4^ cells) and AMB3 + PMMA (0.00367 mmol/10^4^ cells). While the phosphate concentrations per cell constantly rose with higher LAMB concentrations, the phosphate concentrations per cell showed a bell-shaped curve with rising VOR concentrations. Consequently, only VOR2 + PMMA and AMB3 + PMMA revealed significantly higher concentrations compared to NC + PMMA and OC + PMMA, as shown in Figure 6b.

#### 2.2.6. Alkaline Phosphatase Concentration in Supernatants

**Figure 7:**
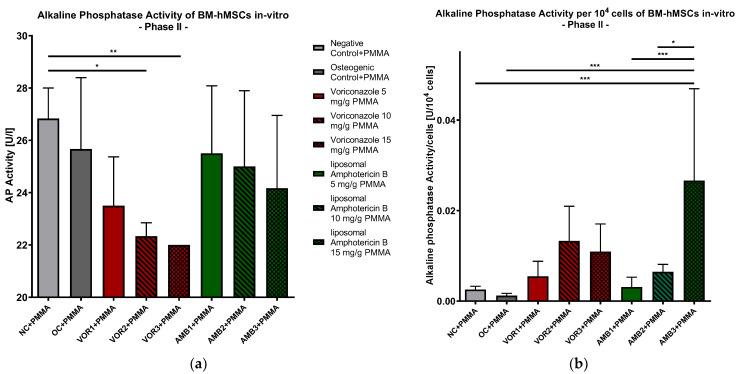
(**a**) Alkaline phosphatase activity in supernatants of BM-hMSC in vitro. Mean alkaline phosphatase activity per group of BM-hMSCs in vitro in Part II in U/l. n = 6. (**b**) Alkaline phosphatase activity per 10^4^ cells in supernatants of BM-hMSC in vitro. Mean alkaline phosphatase activity per group of BM-hMSCs in vitro in Part II normed per 10^4^ cells. n ≥ 4. A 0.5 g PMMA cement chip was placed in the center of every dish. Fungicides were admixed to PMMA in the concentrations displayed in the middle. The error bars show the error of the mean. Significances are indicated by stars; bars under the stars indicate the compared group for each significance. *: *p* < 0.05, **: *p* < 0.01, ***: *p* < 0.001.

Concerning ALP activity, it was slightly but not significantly lower in all fungicide probes in comparison to OC + PMMA, with a continuously decreasing activity when fungicide concentration was increased. Significant differences were detected between NC + PMMA and VOR2 + PMMA (*p* < 0.05), respectively, and VOR3 + PMMA (*p* < 0.01). The results are displayed in Figure 7a.

Norming ALP activity for 10^4^ cells revealed similar results compared to calcium and phosphate concentrations per 10^4^ cells. We detected significantly higher ALP activity in the AMB3 + PMMA group compared to NC + PMMA (*p* < 0.001), OC + PMMA (*p* < 0.001), AMB1 + PMMA (*p* < 0.001), and AMB2 + PMMA (*p* = 0.0192). While bell-shaped ALP activity per cell is visible with increasing VOR concentrations, ALP activity per cell constantly rises in groups with higher LAMB concentrations, as shown in Figure 7b.

#### 2.2.7. Effects of PMMA

**Figure 8:**
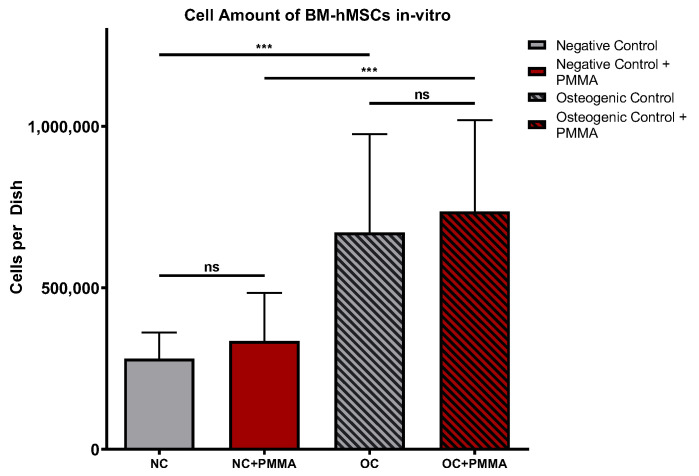
Cells per dish. Mean cell amount per group of BM-hMSCs in vitro assessed by DAPI staining. In NC + PMMA and OC + PMMA a 0.5 g PMMA cement chip was placed in the center of every dish. The error bars show the error of the mean. n ≥ 20. ns: no significance; ***: *p* < 0.001.

To evaluate the effects of PMMA, we compared the control groups of Part I and Part II in terms of the DAPI cell count, ^99m^Tc-HDP uptake, and ^99m^Tc-HDP uptake per cell with each other. A comparison of the cell counts is displayed in Figure 8. There was no significant difference in the cell counts between NC and NC + PMMA, respectively, and OC and OC + PMMA. The amounts of cells in osteogenic groups were significantly higher than in the corresponding negative controls (*p* < 0.001).

**Figure 9:**
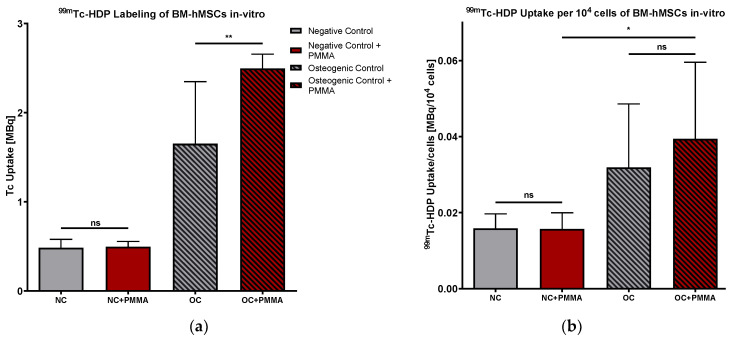
(**a**) ^99m^Tc-HDP uptake in MBq. Mean ^99m^Tc-HDP uptake per group of BM-hMSCs in vitro. n = 6 (**b**) ^99m^Tc-HDP uptake in MBq per 10^4^ cells. Mean ^99m^Tc-HDP uptake per group of BM-hMSCs in vitro was normed for 10^4^ cells. n ≥ 4. In NC + PMMA and OC + PMMA, a 0.5 g PMMA cement chip was placed in the center of every dish. The error bars show the error of the mean. Significances are indicated by stars; bars under the stars indicate the compared group for each significance. ns: not significant, *: *p* < 0.05; **: *p* < 0.01.

^99m^The Tc-HDP uptake is displayed in Figure 9a. Our results revealed no difference between NC and NC + PMMA, while ^99m^Tc-HDP uptake was significantly higher in OC + PMMA compared to OC (*p* < 0.01).

Putting these results together, we calculated the ^99m^Tc-HDP uptake per 10^4^ cells, as displayed in Figure 9b. Comparing ^99m^the Tc-HDP uptake per cell of NC with NC + PMMA and OC with OC + PMMA, our results did not reveal any significant difference. Comparing NC + PMMA with OC + PMMA, there was a significantly higher ^99m^Tc-HDP uptake per 10^4^ cells visible in OC + PMMA than NC + PMMA (*p* < 0.05).

## 3. Discussion

### 3.1. Part I

In comparison to all of the other anti-fungal drugs, VOR negatively affected the osteogenic activity in both concentrations, while there was no negative or positive impact of both LAMB and FLU concentrations upon the osteogenic differentiation potential of BM-hMSC in vitro. Although high-dose concentrations of fungicides revealed some negative side effects in clinical trials, we showed that there are no negative effects of higher fungicide concentrations compared to the corresponding low-dose fungicide. Besides VOR and LAMB, FLU is one of the most common fungicides. Due to the lack of approval for FLU powder for use in humans, its bad mechanical strength, and its inhomogeneous drug release if added as a fluid to PMMA, we decided to only examine FLU in Part I. To measure osteogenesis under the highest possible FLU concentration, we used a concentration of 140 µg/mL as high-dose FLU, although this concentration can only be reached by local administration without leading to systemic side effects. Even at a 15-times higher FLU concentration than the standard systemic treatment serum concentration, there were no negative effects on osteogenesis detected compared to low-dose FLU. This is in line with a study by Allen et al., in which they revealed no negative effects of FLU in concentrations of 15 and 200 µg/mL on osteogenesis and cell counts [48].

According to the cell counts, our data reported significantly higher cell counts in all osteogenic groups without any effects of every anti-fungal drug, both in high- and low-dose concentrations, on cell proliferation. These results are in line with previously published studies, which did not reveal any toxic effects of the tested fungicides in our Part I concentrations [2,17,34,48,49]. Higher cell counts in osteogenic groups are most likely due to dexamethasone in the osteogenic differentiation medium. According to studies by Wang et al. and Both et al., dexamethasone promotes the proliferation of MSC in nmol/L concentrations, as used in this study [50,51]. According to Oshina et al., dexamethasone promotes cell proliferation by enhancing the expression of pro-proliferative gene expression, like FGFR-1 [52].

Although the ^99m^Tc-HDP uptake per cell showed no significant difference between each group, both VOR groups showed a slight but not significantly lower ^99m^Tc-HDP uptake per cell ratio than OC and other fungicide groups. This quotient shows that lower osteogenesis in BM-hMSC treated with VOR is most likely caused by a lower osteogenic activity of the cells and not by reduced cell counts. In principle, there are two possible mechanisms of lower osteogenesis in VOR-treated BM-hMSC. On the one hand, it is conceivable that lower osteogenic differentiation rates of BM-hMSC, resulting in lower osteoblast counts, cause lower osteogenesis. On the other hand, osteogenic differentiation rates could be normal, but the activity of osteoblasts could be impaired. Both mechanisms result in impaired hydroxyapatite production. These results are in line with a publication by Schmidt et al., as they showed negative effects of VOR on cell counts at concentrations of 100 µg/mL, which was 20 times higher than the systemic high-dose VOR concentration we tested [2]. Which mechanism or whether a combination of reduced osteoblast differentiation or osteoblast activity is underlying the reduced hydroxyapatite production was never published, and this should be addressed in further studies.

### 3.2. Part II

#### 3.2.1. Effects of PMMA on the Osteogenic Potential of BM-hMSC

To simulate the clinical reality where anti-mycotic drugs are delivered using PMMA spacers and to distinguish whether our results are affected by PMMA as well, we compared the results of the control groups in Parts I and II with each other. The comparison of the ^99m^Tc-HDP uptake of NC and NC + PMMA revealed similar results, as displayed in Figure 9a. This shows that PMMA does not affect the binding of ^99m^Tc-HDP to hydroxyapatite. This result is in line with prior studies, which showed that ^99m^Tc-HDP binds in a highly specific manner to hydroxyapatite [45,46,47]. Proof of this principle is fundamental for the understanding that ^99m^Tc-HDP uptake is directly proportional to hydroxyapatite concentrations in the probes, and consequently, is a reliable and sensitive method for accessing osteogenesis [47].

Comparing cell amounts of NC and NC + PMMA, we detected no significant difference between the groups. This concurs with findings from the studies of Bastidas-Coral et al. and Saskianti et al., which found that PMMA is not cytotoxic to human stem cells and osteoblasts [41,42]. Bastidas-Coral et al. investigated the effects of PMMA on the osteogenic potential of human adipose stem cells and primary osteoblasts and revealed no adverse effects of PMMA on the osteogenic potential of both cell lines in vitro [41]. Moreover, they showed that PMMA is not cytotoxic to these cell lines [41]. Saskianti et al. demonstrated that PMMA is not cytotoxic to human stem cells and osteoblasts in vitro [42].

Focusing on the osteogenic potential of BM-hMSCs, we detected a significant hydroxyapatite depletion in both osteogenic controls. Comparing the ^99m^Tc-HDP uptakes in osteogenic groups with each other, the ^99m^Tc-HDP uptake in OC + PMMA was significantly higher than in OC, while cell counts did not differ significantly from each other. Consequently, we detected a slightly but not significantly higher ^99m^Tc-HDP uptake per cell quotient in OC + PMMA than in OC. The literature about the effects of PMMA on the osteogenic potential is controversial [38,39,40,41,42,43,44]. Some authors postulate adverse effects of PMMA on the osteogenic potential caused by cytotoxic effects of non-reacted monomers eluted by PMMA or local high temperatures caused by an exothermic reaction [38,39,40]. On the other hand, several studies have shown no cytotoxic effects of PMMA on osteoblasts and stem cells [41,42,43,44]. Although there are controversial effects of PMMA on the osteogenic response reported, we did not find studies that reported a positive impact of PMMA on osteogenesis. Higher ^99m^Tc-HDP uptake in OC + PMMA could be a result of a higher cell density. In PMMA groups, the surface of the dishes was 1.5 cm^2^ smaller because of the PMMA chip placed in the middle of the dish. As a consequence, the surface that was accessible for cell growth was approximately 18% smaller. It is well known that different cell confluences lead to different effects [43,53,54]. Although higher cell concentrations can lead to overgrowth and stop proliferation, cell counts should not be too small because cells need contact with each other for the stimulation of differentiation and proliferation [43,54]. We postulate that the higher ^99m^Tc-HDP uptake in OC + PMMA may be caused by higher hydroxyapatite production due to better cell–cell interactions. Nevertheless, it remains unclear whether more BM-hMSC differentiated into osteoblasts or hydroxyapatite production by osteoblasts was higher. Further experiments should be performed to determine the differentiation rates of BM-hMSC and hydroxyapatite production in osteoblasts.

#### 3.2.2. Effects of Anti-Mycotic Drugs on the Osteogenic Potential of BM-hMSC

PMMA cement with low-dose fungicides is used for definitive fixation of prosthetic joints or as a compound osteosynthesis for large-sized bone defects [18,19,21,22,55]. The purpose behind fixation cement or compound osteosynthesis is the stabilization of prosthesis or osteosynthesis for as long as possible. Due to this, it is most important that added fungicides do not impair mechanical stability [18,21,22,26]. Therefore, maximum fungicide concentrations are low. Mixtures with up to approximately 10% drug powder fraction are still stable enough to fulfil European ISO norms [26]. At this point, it is important to mention that drug powders contain a small amount of drug and several different additives. As a consequence, drug concentration is limited to about 5 mg/g of PMMA [18,21,26]. In a recent study by Krampitz et al., VOR-loaded PMMA cement was tested regarding its mechanical characteristics at concentrations of 5 mg/g and 15 mg/g [26]. They found out that VOR impairs dose-dependent compressive strength and bending resistance [26]. Although VOR impaired mechanical strength, PMMA loaded with 5 mg/g VOR was still stable enough to fulfil the European ISO norms [26]. Czuban et al. tested different Amphotericin B formulations and concentrations admixed to PMMA regarding mechanical strength and porosity [27]. They showed that LAMB, like VOR, impairs mechanical strength and increases dose-dependent porosity [27]. Moreover, added drugs should be heat-resistant and oxidation-resistant due to the exothermic oxidation of PMMA [18,21,22]. In order to homogenize the dilution of the drug, it must be mixed as powder with PMMA powder because of the inhomogeneous dilution of fluids in PMMA [18,21,22]. Consequently, only LAMB and VOR are recommended for admixing to PMMA. The release of bioactive VOR from PMMA was proven by Miller [56], while the bioactivity of LAMB was proven by Cunningham et al. and Czuban et al. [27,57]. In all three studies, a dose-dependent drug release was detected for VOR and LAMB-loaded PMMA [27,56,57].

PMMA cement with high-dose fungicides is used to deliver high drug concentrations at infection sites, and it is either removed or replaced after several days to weeks [7,12,17,19]. The purpose of these spacers is not to provide mechanical stability, and consequently, it is possible to mix more than 10% drug powder with PMMA [26]. The maximum concentration is only limited by local or systemic side effects or the maximum admixable drug powder, which still enables polymerization of the cement. In the past, 10 mg of VOR and LAMB, as well as 15 mg of VOR per g of PMMA, were already tested for their mechanical stability [26], and in this study, we present the first in vitro data concerning osteogenesis and proliferation.

Our data clearly show that all fungicide-loaded cement discs reduce dose-dependency on the cell amount in the dishes. According to these results, VOR- and LAMB-loaded PMMA chips are cytotoxic, and cytotoxicity increases with increasing fungicide concentrations. These findings concur with studies examining the adverse effects of antimycotic drugs. For VOR, there are a number of known adverse events, including hepatotoxicity and osteomyelitis [58]. Schmidt et al. published a study in 2013 about the dose-dependent cytotoxicity of VOR to mouse fibroblasts and osteoblasts in vitro [2]. According to this study, VOR concentrations of up to 100 µg/mL did not decrease cell growth [2]. Concentrations of 500 to 1000 µg/mL impaired cell growth significantly, but the cells recovered within 7 days after the removal of VOR [2]. Concentrations of 5000 µg/mL or higher resulted in the cell death of osteoblasts [2]. Miller et al. analyzed VOR release from PMMA cement in concentrations of 7.5 mg/g and 15 mg/g PMMA over 30 days [56]. According to this study, the VOR concentration in our study was most probably between 100 and 500 µg/mL in cell culture medium. These concentrations are much higher than serum levels after systemic drug administration but seem to be realistic for local concentrations next to PMMA, and they are the reason for lowered cell counts in every VOR group in Part II of this study.

According to our results of ^99m^Tc-HDP uptake and ^99m^Tc-HDP uptake per cell ratio we detected, that although the total hydroxyapatite production of the cells was significantly lower in all VOR-treated groups, the relative ability of every cell to produce hydroxyapatite was slightly but not significantly higher. These results can be underlined by the results of our supernatant analysis. Because the calcium concentration in supernatants is known as an inverse non-invasive marker of osteogenesis, our results confirm that osteogenesis in VOR2 + PMMA and VOR3 + PMMA is significantly lower than in VOR1 + PMMA [5,59]. Moreover, our results of the phosphate concentration and ALP activity show similar dose-dependent effects of VOR on the osteogenic potential of BM-hMSC. Normed calcium and phosphate concentrations per cell as well as normed ALP activity per cell showed a similar bell-shaped pattern in VOR groups as those that we detected in VOR groups for ^99m^Tc-HDP uptake per cell, and consequently, this strengthened our results about osteogenic potential per cell ratio. This concurs with other research [5,48]. Hofmann et al. reported a significantly higher hydroxyapatite production if VOR was added to cell culture media [5]. Allen et al. published that 15 µg/mL and 200 µg/mL of VOR enhance osteoblastic activity and differentiation [48]. Moreover, they reported higher expression of the osteoblastic cytokine vascular endothelial growth factor (VEGF) and platelet-derived growth factor (PDGF) [48]. In VOR1 + PMMA and VOR2 + PMMA, we detected these positive effects on the osteoblastic activity and differentiation too, which is clearly visible in higher ^99m^Tc-HDP uptake per cell ratios as well as higher phosphate and ALP ratios. But, as already mentioned, VOR has cytotoxic effects as well. We think that VOR1 + PMMA enhancement of osteoblastic activity has a higher impact than cytotoxicity, and consequently, the osteogenic potential is similar to that of OC + PMMA. With rising VOR concentrations, cytotoxicity rises faster than osteoblastic activity, as seen in significantly lower ^99m^Tc-HDP uptake but slightly higher ^99m^Tc-HDP uptake per cell ratio in VOR2 + PMMA than in OC + PMMA. In VOR3 + PMMA, the cytotoxic effects are so high that Voriconazole cannot evolve induction of osteoblastic activity, and consequently, the cell count, ^99m^Tc-HDP uptake, ^99m^Tc-HDP uptake per cell ratio, and phosphate and ALP activity ratios are reduced.

Like VOR, Amphotericin B is cytotoxic and can induce dose-dependent, severe side effects [19,28,30,36,49]. To reduce side effects, Amphotericin B can be encapsulated in liposomes (LAMB) [30,36]. Due to lipid formulation, LAMB extravascular liberation from liposomes is limited, which results in high serum levels and low tissue concentrations [28]. The enhanced permeability of the blood–tissue barrier in infected tissues leads to an accumulation of LAMB at the infection side with exceptionally high local drug concentrations [28,60]. Nevertheless, LAMB still has dose- and time-dependent cytotoxic effects [30,61,62]. Harmsen et al. showed that Amphotericin B is time- and concentration-dependently cytotoxic to osteoblasts in vitro [62]. Larabi et al. compared different Amphotericin B formulations with each other and found out that different lipid formulations have different cytotoxic potentials with the lowest potential of the lipid formulation AmbiSome^®^, which was used in our study [61]. Roberts et al. compared different Amphotericin B formulations in vitro and in vivo regarding cytotoxicity [63]. They showed that LAMB had temporarily cytotoxic effects on mouse osteoblasts in concentrations of 5–1000 µg/mL after 7 days of incubation with full recovery after 3 days without fungicide incubation, while no cytotoxicity of fungicide-loaded PMMA cement chips (concentration 5–20 mg/g PMMA) implanted into rat paraspinal muscle was detected [63]. As a possible reason, they discussed the formation of liposomes of LAMB after release [63]. Cunningham et al. analyzed PMMA cement loaded with LAMB regarding mechanical strength, drug release, and bioactivity of the released drug [57]. They tested LAMB at concentrations of 5 mg/g and 20 mg/g PMMA and found a dose-dependent release profile of still bioactive LAMB [57]. According to Cunningham et al., the LAMB concentration in our study was most likely between 15 µg/mL and 75 µg/mL. Whether these concentrations can induce cytotoxicity in a long-term cell culture, like ours, has not been investigated in the past, but it seems probable that cytotoxic effects will occur with these concentrations in long-term incubations. Comparing the cell counts of LAMB groups in Parts I and II with each other, reduced cell amounts were only detectable in groups with drug release from PMMA. It is most likely that the reduced cell amounts in PMMA groups are due to a combination of cytotoxic effects of PMMA and LAMB, although we did not prove this.

Analyzing the effects of LAMB on the osteogenic potential, LAMB in concentrations of 5 mg/g and 10 mg/g PMMA showed no altered ^99m^Tc-HDP uptake, while the ^99m^Tc-HDP uptake in groups with 15 mg/g LAMB in PMMA was significantly reduced. These results can be underlined by similar results from the supernatant analysis of calcium, phosphate, and ALP activity, although ALP activity results are not significant. Due to different enzyme formations and several specific roles besides osteogenesis ALP activity results can be affected by several other influences, and consequently, it is not surprising that ALP activity results are not as clear as ^99m^Tc-HDP labeling [5]. In comparison to VOR and OC, calcium concentrations were clearly lower in all LAMB groups. This concurs with the findings of Sealy et al., who detected 50–60% lower calcium concentrations in Amphotericin B-loaded PMMA beads [17].

On the other hand, the ^99m^Tc-HDP uptake per 10.000 cells ratio rose with increasing concentrations of LAMB. The same effects were detected in the phosphate concentrations and ALP activity normed for cell count in supernatants. These findings are in line with a study by Skubis et al. in 2017 [64]. They reported enhanced osteogenesis of human adipose-derived mesenchymal stem cells in vitro [64]. Like in VOR groups, we postulate that our results are a combination of pro-osteogenic and cytotoxic effects. We detected with rising LAMB concentrations increasing cytotoxic effects as visible in reduced cell amounts, but rising osteogenic effects as visible in higher ^99m^Tc-HDP uptake per cell ratio, higher phosphate concentrations per cell, and higher ALP activity per cell ratio. Due to a relatively lower impact of LAMB on osteogenesis rather than cell viability, an absolute ^99m^Tc-HDP uptake decline was observed with rising LAMB concentrations.

Although our study shows relevant differences between the impact of VOR and LAMB-loaded PMMA cement on the osteogenic potential of BM-hMSCs in vitro, it is important to mention that the successful treatment of a fungal infection should always be the first aim because any active infection impairs the osteogenic potential of BM-hMSC.

### 3.3. Limitations

A limitation of our study is that all of our experiments were performed in vitro, and therefore, in vivo studies should be conducted to transfer the findings into clinical practice. Systemic effects like enzymatic drug modification and drug depletion in other organs could be a significant factor in vivo while not being applicable in the local administration of drugs. Moreover, drug elimination was not physiologically due to a medium change every 2 to 3 days and different distribution volumes in vivo. Another limitation of this study was the duration of the cell culture. Although 21 days in a cell culture is quite long, it is still shorter than the normal duration of systemic anti-fungal drug therapy and the remaining drug-loaded PMMA spacers at infection sites. Some studies have revealed that the negative effects of fungicide-loaded cement spacers on cell viability could be temporary during fungicide exposure and that cell function could be fully restored after the termination of drug exposure [2,63]. As far as we know, no data concerning the recovery of BM-hMSC regarding cell viability and osteogenic potential have been published. Because we did not analyze the recovery of the cells after the termination of drug exposure, we plan to conduct further research to determine whether the cell viability and osteogenic potential of BM-hMSC can be retrieved after the end of drug exposure. An important part of an organism’s response to implants like PMMA is the formation of a foreign-body-induced membrane [65,66]. To our knowledge, there is no established in vitro model that reflects the presence of a foreign-body-induced membrane. Consequently, we could not evaluate the effects of a foreign-body-induced membrane on our experimental in vitro setting. Further studies should be performed to distinguish whether an impaired osteogenic potential is a consequence of cytotoxic effects, impaired osteogenic differentiation of MSCs, or impaired osteogenic activity of osteoblasts. However, this study is a solid foundation for further in vivo studies to gain a better understanding of the effects of systemic and locally administered fungicides on the osteogenic potential of MSCs.

## 4. Materials and Methods

**Figure 10:**
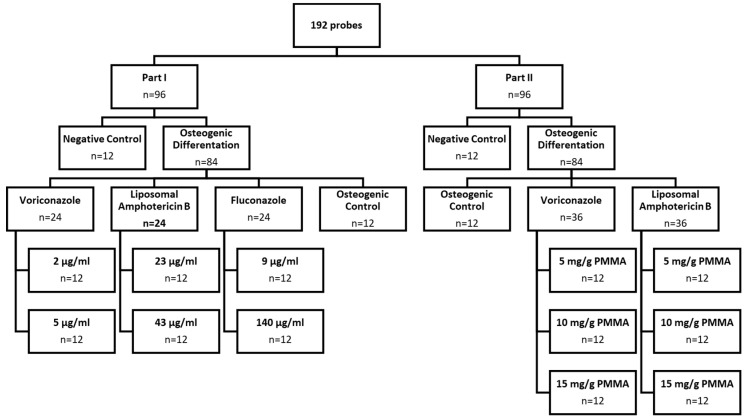
Study design.

At a glance, this study was performed in two parts, as displayed in Figure 10. Part I was performed to investigate the effects of the three most common anti-fungal drugs for systemic therapy, VOR, LAMB, and FLU, each at low-dose and high-dose concentrations. In Part I, we applied fungicides directly to the cell culture medium and assessed the osteogenic potential of BM-hMSC in vitro.

According to clinical studies about the outcomes adjusted to serum concentrations of VOR, we decided to use 2 µg/mL, which is located at the lower end of the recommended target concentration as the low-dose concentration, and 5 µg/mL, which is located at the upper end of the recommended target concentration, because of rising toxic side effects [28,29,67,68,69]. Amphotericin B is the most commonly used anti-fungal polyene with a wide spectrum of side effects, which are significantly lower if Amphotericin B is applied as a liposomal formulation [28,30]. Effective serum concentrations of LAMB were detected at around 23 ± 10 µg/mL [28,36]. dDue to the liposome structure and protein-binding LAMB diffuse variable into different tissues at higher diffusion rates in more permeable tissues [28,35]. This leads to 2–5 times higher concentrations in different tissues [33,34,35]. According to animal studies, the concentration of LAMB in bone marrow is about 40 µg/mL, so we used 23 µg/mL as our low concentration and 43 µg/mL as our high concentration in this study [70]. Standard systemic treatment with FLU results in serum concentrations of 9 µg/mL but can lead up to 92 µg/mL [31,33,37]. In addition to systemic treatment, FLU was mixed with PMMA in several studies [23,71]. In these studies, FLU concentrations of up to 140 µg/mL were detected [23]. Because FLU is only available as a fluid and this would result in inhomogeneous drug release if added to PMMA cement, we decided to use FLU only in Part I of this study. Therefore, we decided to perform Part I experiments with 9 µg/mL as the low-dose concentration and 140 µg/mL as the high-dose concentration, although this high-dose was only reported after local administration of FLU.

In Part II, we evaluated how fungicide-loaded PMMA cement chips affect the osteogenic potential of BM-hMSC in vitro to mimic the situation as it occurs during in-human treatment. Because admixing FLU to PMMA would result in inhomogeneous drug release, we decided to investigate the effects of the two most common fungicides VOR and LAMB, which are both approved by the FDA and EMA as admixes to PMMA [18,21,22]. In line with the recommendations of PRO-IMPLANT FOUNDATION, we evaluated the effects of low-dose fixation cement and high-dose spacer cement, as well as an experimental extended-dose spacer cement [21,22]. The recommended concentrations for VOR and LAMB are identical, so we loaded PMMA chips with VOR or LAMB in the following concentrations: 5 mg/g of PMMA, 10 mg/g of PMMA, and 15 mg/g of PMMA [21,22].

### 4.1. Harvest of BM-hMSC

This study, and especially the surgical harvesting of BM-hMSC, was approved by the Ethics Committee board, University Hospital Heidelberg, Faculty of Medicine, Heidelberg, Germany (No. S-309/2007), on 23 November 2007.

For this study, we used the primary BM-hMSCs of 12 healthy donors (n = 12) only. Six donors were needed for each part of the study. Bone marrow aspirates were obtained from the proximal femoral cavity under general anesthesia during an elective surgical total hip arthroplasty after informed consent was obtained. During preparation of the proximal femoral bone cavity, 10–15 mL of bone marrow was collected into a 20 mL syringe (BD, Heidelberg, Germany) containing 1000 IU of heparin (Braun, Melsungen, Germany). Individual samples were diluted 1:1 with a phosphate-buffered saline (PBS) (Gibco, Frankfurt, Germany) and washed twice with PBS. For isolation of the mononuclear cell fraction, we used the Ficoll^®^ gradient centrifugation (Ficoll-Paque-PLUS; Cytiva, Freiburg, Germany) as previously described [52,72,73,74]. The interphase section of Ficoll^®^ gradient was extracted, and mononuclear cells were plated in T-150 polystyrene tissue culture flasks (Falcon, Kaiserslautern, Germany) at a density of 5 × 10^5^/cm^2^ and cultured in a humidified 5% CO_2_ atmosphere at 37 °C in high-glucose Dulbecco’s modified Eagle’s medium (DMEM-HG, Gibco, Germany) containing 10% heat-inactivated (56 °C, 30 min) fetal bovine serum (FCS) (Sigma, Schnelldorf, Germany) and 1% Penicillin/Streptomycin (Sigma). Before cell seeding, flasks were coated with 0.5% gelatine (Sigma) in PBS for 30 min.

After 48 h, all nonadherent cells were removed by washing with PBS. According to Dominici et al., plastic adherent cells were defined as BM-hMSC [75]. Media were changed three times a week (every 2–3 days). At 90% confluence, cells were trypsinized (Trypsin-EDTA, Sigma) and stored as P1 cells for further experiments in liquid nitrogen. Each 1 mL aliquot contained 1 × 10^6^ cells in 50% FCS, 40% DMEM, and 10% Dimethyl sulfoxide (DMSO) (Sigma).

### 4.2. Expansion of BM-hMSC

Before osteogenic differentiation, P1 BM-hMSC of 12 donors was defrosted, and 250,000 cells were seeded in gelatine-coated T150 flasks (Falcon) and cultured for 10 days in a humidified 5% CO_2_ atmosphere at 37 °C. Expansion media DMEM low glucose (DMEM LG) with 10% FCS and 1% Penicillin/Streptomycin were changed every second day. At a confluence of 80–90%, which was reached after approximately 5 days, cells were trypsinized and used for differentiation.

### 4.3. Osteogenic Differentation of BM-hMSC

Cells from every donor (n = 12) were seeded in duplicates into 16 35 mm flat-bottom Petri dishes (Corning, Kaiserslatuern, Germany), each at a density of 10^4^ cells/cm^2^. n = 96 probes were used for each part of the study, so n = 192 were used in total. During 21 days of differentiation, we used DMEM LG with 10% FCS with media change three times a week (every 2–3 days). Osteogenic differentiation was performed using the following standard osteogenic supplements: 100 nM dexamethasone, 50 µM L-ascorbic acid, and 10 nM glycerol phosphate.

### 4.4. Preparation of the Dishes for Part I

For Part I of our experiment, each dish received its corresponding differentiation medium, as demonstrated in Table 1. One group was treated as a negative control (control DMEM, NC) and received only DMEM LG + 10% FCS without osteogenic differentiation supplements and no anti-fungal drug. A second control group (osteogenic control, OC) received osteogenic differentiation medium (DMEM LG + 10% FCS + 100 nM dexamethasone, 50 µM L-ascorbic acid + 10 nM glycerol phosphate) but no anti-fungal drug. Each of the three fungicides, VOR (VFEND, Pfizer Europe MA EEIG, Brussels, Belgium), LAMB (AmBisome liposomal, Gilead Sciences GmbH, Martinsried, Germany), and FLU (Fluconazol Kabi, Fresenius Kabi Deutschland GmbH, Bad Homburg, Germany), was tested in low- and high-dose concentrations.

### 4.5. Preparation of the Dishes for Part II

Part II of the study evaluated the effects of fungicides eluted from fungicide-loaded PMMA on the osteogenic response of BM-hMSC. Before seeding the cells, a 0.5 g PMMA cement chip (PALACOS^®^ R, Heraeus Medical, Frankfurt, Germany) was placed at the center of every cell culture dish. PMMA was polymerized as recommended in the instruction for use. Components were mixed by hand for 30 s and PMMA was polymerized for 15 min under sterile conditions and at room temperature. Antimycotics were added as powder to the powder component of the cement. Like in Part I, one group was treated as a negative control with a non-loaded PMMA chip (control DMEM + PMMA, NC + PMMA) and received only DMEM LG + 10% FCS without osteogenic differentiation supplements. A second control group with a non-loaded PMMA chip (osteogenic control + PMMA, OC + PMMA) received the same osteogenic differentiation medium as in Part I. For fungicide groups, fungicides were solved in PMMA by mixing the drug powder with the PMMA powder component, and 0.5 g of PMMA cement chips were placed in the center of every cell culture dish. For Part II fungicides, we used the same drugs as in Part I (VOR: VFEND, Pfizer Europe MA EEIG, Brussels, Belgium, LAMB: AmBisome liposomal, Gilead Sciences GmbH, Martinsried, Germany). The study design for Part II is visualized in Table 2.

Cells were cultured for 21 days in a humidified 5% CO_2_ atmosphere at 37 °C with media change three times a week (every 2–3 days). After 21 days, the cell cultures were terminated. Supernatants were collected from Part II cells and stored in 1 mL aliquots at −20 °C for further analysis. For ^99m^Tc-HDP labeling, cells were washed twice with PBS, followed by air-drying under a cell culture hood. For the DAPI cell count, cell cultures were directly fixated with 70% ethanol (Sigma) at room temperature for 10 min and stained as described below.

### 4.6. DAPI Staining and Cell Count

After the termination of cell culture, half of the samples (n = 6 per group) were used for the DAPI cell count. Fixated cells were washed with PBS twice and stained with DAPI (1:1000 in PBS) for 5 min followed by washing cells twice with PBS. Stained cells were counted under a microscope (Leica DMi8, Leica, Wetzlar, Germany). For each sample, five random vision fields were selected, and cells were counted within these fields. Automated cell counting was performed with the software “CellProfiler” (Broad Institute, URL: https://cellprofiler.org, version 4.2.4 accessed on 10 October 2022) and manually checked for correct counting. The counts were assessed for normal distribution using the Kolmogorov–Smirnov test. The mean values were calculated to norm other results to cell counts. Regarding the specifications of the microscope and magnification, each vision field has an area of 270.835 µm^2^. The absolute cell count was calculated by multiplying the vision field with the entire cell culture dish.

### 4.7. ^99m^Tc-HDP Labeling and Analysis

Cells were labeled by adding 5 MBq ^99m^Tc-HDP in 1 mL of NaCl 0.9% solution to each dish. IFor incubation, dishes were stored at room temperature for 15 min before the remaining solved ^99m^Tc-HDP was removed, and the dishes were washed twice in PBS to remove the unbound radiotracer. The dishes were then analyzed using the dose calibrator (Activimeter ISOMED 1010, Nuklearmedizintechnik Dresden, Dresden, Germany) to exactly determine the amount of bound tracer. Therefore, dishes were placed directly in the detection chamber. Gamma counts were detected for 5 s for each dish. ^99m^Tc-HDP uptake was reported in activity of bounded ^99m^Tc in MBq. Further analysis results were normed for 10^4^ cells.

### 4.8. Analysis of Supernatants

n = 6 aliquots per group filled with supernatants of Part II (96 samples in total) were analyzed for the three osteogenic markers calcium and phosphate concentration, as well as ALP activity by the central laboratory of the University Hospital Heidelberg. As ALP is one of the key enzymes in the formation of extracellular bone matrix, measuring ALP concentration in supernatants is a well-established indirect method of evaluating osteogenic potential [5,44,48,76,77]. Phosphate is a synthesis product of ALP [76]. As ALP is an established indirect marker for osteogenesis, phosphate levels rise with higher ALP activity and indicate higher osteogenesis [5,76]. Measuring supernatant calcium concentrations is another established inverse non-invasive marker of osteogenesis because supernatant calcium concentrations decline with rising osteogenic potential [5,62]. Calcium and phosphate concentrations and ALP activity were measured photometrically using Siemens Dimension EXL200 (Siemens AG, Erlangen, Germany) with validated standards for analyzing cell culture media. Further analysis results were normed for 10^4^ cells.

### 4.9. Statistics

For the statistical analysis, all of the results were tested for normal distribution using the Kolmogorov–Smirnov test first. Then, an ANOVA analysis with Bonferroni post-hoc testing was performed to determine statistical significances between the groups. ^99m^Tc-HDP labeling was analyzed for total gamma counts and normed for 10^4^ cells to evaluate the osteogenic potential per cell. A statistical analysis of the supernatants was performed for concentration/activity per dish and per 10^4^ cells. GraphPad Prism (GraphPad Software, Boston, MA, USA), Version 6, was used for statistical analysis and visualization. Statistical significance was set to *p* ≤ 0.05.

## 5. Conclusions

In general, our results revealed that BM-hMSCs treated with fungicides in all tested concentrations have a significant osteogenic response in comparison to the corresponding negative control under systemic and PMMA-delivered fungicide conditions. Although osteogenesis was detected in all osteogenic groups, there were significant differences in the dimension of osteogenesis in different fungicide groups.

As a conclusion of Part I, we revealed that both high- and low-dose VOR concentrations negatively and significantly affect the osteogenic potential of BM-hMSC in vitro, while there was no detectable effect on the cell count in both concentrations. Regarding our data, it remains unclear whether lower osteogenic activity in BM-hMSC treated with VOR is caused by less differentiation of BM-hMSC into osteoblasts or less activity of osteoblasts. Both concentrations of LAMB and FLU added to a cell culture medium do not affect cell counts or the osteogenic potential of BM-hMSC in vitro.

As a conclusion of Part II, we clearly detected dose-dependent effects of fungicides delivered by fungicide-loaded PMMA spacers. Our data revealed that negative effects on the osteogenic potential due to the cytotoxicity of fungicides can be compensated by BM-hMSC in low-dose VOR (VOR1 + PMMA) and LAMB (AMB1 + PMMA), as they are frequently used in PMMA fixation cement. We found that high-dose LAMB (AMB2 + PMMA) does not impair osteogenic potential, while there is an impairment of the osteogenic potential detectable in high-dose VOR (VOR2 + PMMA), as both are used in PMMA-spacers. PMMA cement with extended fungicide doses (VOR3 + PMMA and AMB3 + PMMA) affects the osteogenic potential of BM-hMSC negatively, which is most probably due to cytotoxic effects.

Because this study was performed in vitro, it is necessary to prove the findings in vivo before clinicians overthink their performance. It is remarkable that we found no in vivo study about the effects of fungicide-loaded PMMA on osteogenesis. Nevertheless, according to this study, the correct dosage and the correct choice of fungicides are very important. Besides the systemic side effects, clinicians should be aware that fungicides can affect the osteogenic potential of BM-hMSC, as we detected for both systemic VOR concentrations (VOR1 and VOR2). The importance of the correct dosage and the right choice of fungicide grows immensely if fungicides are used in local antimycotic drug therapy delivered by PMMA. If our results can be proven in vivo, clinicians should use low-dose VOR or low- or high-dose LAMB in PMMA for local anti-fungal drug therapy whenever possible to avoid cytotoxicity and reduce the osteogenic potential. Even if the stability of PMMA spacers could be a minor characteristic in some cases, fungicide concentration should not be increased even more to avoid the impairment of osteogenic potential. Nevertheless, fungal eradication is still the most important because persistent fungal infections often result in the loss of limbs or a patient’s death.

## Figures and Tables

**Table 1:** Study design of Part I. X: method on the left was performed in this group.

Group	Negative Control	Osteogenic Control	Voriconazole	Liposomal Amphotericin B	Fluconazole
Abbreviation	NC	OC	VOR1 VOR2	AMB1 AMB2	FLU1 FLU2
Fungicide Concentration	-	-	2 µg/mL 5 µg/mL	23 µg/mL 43 µg/mL	9 µg/mL 140 µg/mL
Medium	DMEM LG + FCS	DMEM LG + FCS	DMEM LG + FCS	DMEM LG + FCS	DMEM LG + FCS
Osteogenic Differentiation - 100 nM dexamethasone - 50 µM L ascorbic acid - 10 nM glycerol phosphate	-	X	X	X	X
^99m^Tc-HDP Labeling	X	X	X	X	X
DAPI/Cell Count	X	X	X	X	X

**Table 2:** Study design of Part II. X: method on the left was performed in this group.

Group	Negative Control	Osteogenic Control	Voriconazole	Liposomal Amphotericin B
Abbreviation	NC	OC	VOR1 + PMMA VOR2 + PMMA VOR3 + PMMA	AMB1 + PMMA AMB2 + PMMA AMB3 + PMMA
Fungicide Concentration per g PMMA	-	-	5 mg/g PMMA 10 mg/g PMMA 15 mg/g PMMA	5 mg/g PMMA 10 mg/g PMMA 15 mg/g PMMA
Medium	DMEM LG + FCS	DMEM LG + FCS	DMEM LG + FCS	DMEM LG + FCS
Osteogenic Differentiation - 100 nM dexamethasone - 50 µM L ascorbic acid - 10 nM glycerol phosphate	-	X	X	X
^99m^Tc-HDP Labeling	X	X	X	X
DAPI/Cell Count	X	X	X	X
Supernatant Analysis - Calcium Concentration - Phosphate Concentration - ALP Activity	X	X	X	X

## Data Availability

The raw data supporting the conclusions of this article will be made available by the authors on request.
